# Burnout among nursing students: a mixed method study

**DOI:** 10.17533/udea.iee.v38n1e07

**Published:** 2020-02-26

**Authors:** Maria José Quina Galdino, Laio Preslis Brando Matos de Almeida, Luiza Ferreira Rigonatti da Silva, Edivaldo Cremer, Alessandro Rolim Scholze, Júlia Trevisan Martins, Maria do Carmo Fernandez Lourenço Haddad

**Affiliations:** 1 Nurse, Ph.D. Professor, Universidade Estadual do Norte do Paraná (UENP), Bandeirantes, Brasil. Email: mariagaldino@uenp.edu.br Universidade Estadual do Norte do Paraná Universidade Estadual do Norte do Paraná (UENP) Bandeirantes Brazil mariagaldino@uenp.edu.br; 2 Nursing Graduating. Universidade Estadual do Norte do Paraná UENP, Bandeirantes, Brasil. Email: laioalmeida34@gmail.com Universidade Estadual do Norte do Paraná Universidade Estadual do Norte do Paraná UENP Bandeirantes Brazil laioalmeida34@gmail.com; 3 Nursing Graduating. Universidade Estadual do Norte do Paraná UENP, Bandeirantes, Brasil. Email: luziaferreirarigonatti@gmail.com Universidade Estadual do Norte do Paraná Universidade Estadual do Norte do Paraná UENP Bandeirantes Brazil luziaferreirarigonatti@gmail.com; 4 Nurse, Ph.D. Professor, Universidade Estadual do Norte do Paraná UENP, Bandeirantes, Brasil. Email: edvaldocremer@uenp.edu.br Universidade Estadual do Norte do Paraná Universidade Estadual do Norte do Paraná UENP Bandeirantes Brazil edvaldocremer@uenp.edu.br; 5 Nurse, Ph.D Student. Professor, Universidade Estadual do Norte do Paraná UENP, Bandeirantes, Brasil. Email: scholze@uenp.edu.br Universidade Estadual do Norte do Paraná Universidade Estadual do Norte do Paraná UENP Bandeirantes Brazil scholze@uenp.edu.br; 6 Nurse, Ph.D. Professor, Universidade Estadual de Londrina (UEL), Londrina, Brasil. Email: jtmartins@uel.br Universidade Estadual de Londrina Universidade Estadual de Londrina (UEL) Londrina Brazil jtmartins@uel.br; 7 Nurse, Ph.D. Professor, Universidade Estadual de Londrina UEL, Londrina, Brasil. Email: carmohaddad@gmail.com Universidade Estadual de Londrina Universidade Estadual de Londrina UEL Londrina Brazil carmohaddad@gmail.com

**Keywords:** burnout, profesional, students, nursing, surveys and questionnaires., agotamiento professional, estudiantes de enfermería, encuestas y cuestionarios.

## Abstract

**Objective.:**

Investigate the burnout syndrome among undergraduate students in nursing.

**Methods.:**

Explanatory sequential mixed method study conducted at a public university in Brazil. Of the 119 nursing students, 114 consented to participate and answered a questionnaire composed of sociodemographic, academic variables, and the Maslach Burnout Inventory - Student Survey, which were analyzed by multiple linear regression. The participants of the quantitative phase with the indicative / risk of burnout were interviewed individually (*n*=21) to provide an in-depth understanding of the students' experiences regarding the dimensions of the syndrome, whose statements were analyzed by the Collective Subject Discourse.

**Results.:**

The prevalence of burnout syndrome was 10.5% among the surveyed. The more advanced the school year, the higher were the exhaustion (*p*=0.003), depersonalization (*p*<0.001) and low academic effectiveness (*p*=0.012) scores. Students with a higher workload of assignments also had higher scores of exhaustion (p=0.001), depersonalization (*p*<0.001) and academic (in)effectiveness (*p*=0.042). Dissatisfaction with the course was related to higher exhaustion (*p*=0.049) and depersonalization (*p*=0.001). The collective speeches showed the daily demands of the course, considered as intense, producing overload and exhaustion, which produced symptoms of physical and mental illness. Thus, there was the student's distancing from the course activities, as a defensive attitude, which culminated in feelings of incompetence and frustration.

**Conclusion.:**

The occurrence of burnout syndrome dimensions among nursing students was related to the activities of academic daily life. It is urgent to invest in health promotion and prevention actions of these individuals in the university context.

## Resumen

**Objetivo.:**

Estudiar el síndrome de *Burnout* entre estudiantes de enfermería.

**Métodos.:**

Estudio de método mixto secuencial explicativo realizado en una universidad pública de Brasil. De los 119 alumnos de enfermería, 114 consentieron en participar y respondieron un cuestionario compuesto por variables sociodemográficas, académicas y el *Maslach Burnout Inventory* ^TM^ *- Student Survey*. Los participantes de la fase cuantitativa con el indicador de riesgo de *burnout* se entrevistaron individualmente (*n*=21) para proporcionar una comprensión profunda de las experiencias de los estudiantes acerca de las dimensiones del síndrome**,** cuyos testimonios se analizaron con la técnica de Discurso del Sujeto Colectivo*.*

**Resultados.:**

La prevalencia de sindrome de *burnout* fue de 10.5% entre los investigados. Cuanto más avanzado el año de estudio del alumno, eran mayores los puntajes de agotamiento (*p*=0.003), despersonalización (*p*<0.001) y baja eficacia académica (*p*=0.012). Los estudiantes con mayor carga horaria de asignaturas también presentaron mayores puntuaciones de agotamiento (*p*=0.001), despersonalización (*p*<0.001) e (in)eficacia académica (*p*=0.042). La insatisfacción con el curso se relacionó con un mayor agotamiento (*p*=0.049) y despersonalización (*p*=0.001). En los discursos colectivos se evidenciaron las exigencias cotidianas del curso, consideradas como intensas, generadoras de sobrecarga y agotamiento, que producían síntomas de enfermedad física y mental. Por lo tanto, como una actitud defensiva, hubo distanciamiento del estudiante de las actividades del curso que culminó en sentimientos de incompetencia y frustración.

**Conclusión.:**

La aparición de las dimensiones del síndrome de *burnout* entre los estudiantes de enfermería estuvo relacionada con las actividades de la vida diaria académica. Es urgente establecer acciones de promoción y prevención en salud para estos individuos en el contexto de la universidad.

## Introduction

Significant levels of psychological disorders and psychological distress among higher education students have been reported worldwide, given that during these years there is a peak in prevalence of many mental disorders, particularly major depressive disorder (18.5% to 21.2%), generalized anxiety disorder ( 18.6% to 16.7%) and drug use disorder (45.9% to 59.8%).(1) This high prevalence is significant both for the suffering it causes in a period of major life transition and for the substantial impairment in academic performance,(2) as well as suicidal thoughts and behaviors.(3) In this context, a psychological disorder widely used as an indicator of mental health in this population is the burnout syndrome or “academic burnout”, which occurs when the individual's defense strategies are ineffective in coping with stress and overload from the academic environment. affecting biopsychosocial health.(4,5)

Burnout syndrome among students is characterized by a sense of overload, called emotional exhaustion; distanced and defensive posture in relation to the study, the teachers and the classmates, a process known as depersonalization; and the perception of being an incompetent student who typifies the reduced academic effectiveness.(4,6) In nursing, the syndrome has already been demonstrated among students,(7,8) residents,(9) master's and doctoral students(10) due to the various demands that exist in the training processHowever, among the students, the factors associated with burnout syndrome were not explored, neither by using a mixed approach that encompasses representativeness and the truth implicit in subjectivity. 

Among the difficulties experienced by nursing students during their academic training are: the study schemes; fulfillment of activities in a tight deadline; time management; the power relationship between teacher-student; the practices in health services; direct contact with the patient, their suffering and the possibility of their death; ethical dilemmas; fear of making mistakes and dealing with internal demands,(11,12) situations that require student adaptation or may overload the student and predispose to burnout syndrome. That said, burnout syndrome should not be considered an individual disorder, attributed solely to the student who becomes ill, the institution's responsibility should be considered in the academic training process that may lead to the student's illness.(13) 

In this context, analyzing burnout syndrome among undergraduate nursing students may provide support for managers to implement prevention and management strategies in relation to the syndrome, in order to ensure health and well-being during the professional training process, as well as providing training for nurses engaged and prepared to provide quality care. Thus, this study aimed to investigate the burnout syndrome among undergraduate nursing students.

## Methods

This is a mixed method, explanatory sequential study that combined quantitative and qualitative approaches. In this design, qualitative data (interviews) provide a deeper picture of the quantitative data (questionnaires) initially obtained.(14) Due to the complementarity character, this integration makes it possible to neutralize the limitations and weaknesses of each research approach.(15)

The research was conducted with undergraduate nursing students from a public university in the southern region of Brazil, since they met the criterion of not being away from academic activities by leaves of absence. The course form generalist nurses through a 4490-hour curriculum matrix (annual series and minimum duration of five years full-time), which inserts the student in theoretical, laboratory and clinical practices in health services.

The quantitative phase was held from July to August 2016, in which all 119 enrolled in the course were invited to participate in the study. Data collection was carried out in the classroom, where participants were informed about the study and the questionnaire was distributed to 114 (95.8% of total) students who agreed to participate voluntarily, of which 31 were enrolled in the first year, 23 in the second, 17 in the third, 21 in the fourth and 25 in the fifth year.

To obtain the data, the authors developed a semi-structured questionnaire containing sociodemographic variables (gender, age, marital status, having children and those with whom they live), lifestyle (physical and leisure) and academic (school year, number of subjects/ lectures attended, total workload assigned to the subjects /lectures, hours devoted to extra-class studies, receiving undergraduate or extension scholarships and satisfaction with the course) for the characterization of the participants. This questionnaire was submitted to a refinement through the evaluation of 5 (five) PhD teachers with experience in occupational health and teaching, who considered that these items were sufficient to reach the objective of the study.

The burnout syndrome was assessed by the Maslach Burnout InventoryTM - Student Survey (MBI-SS), an instrument developed by Schaufeli and colleagues that specifically evaluates burnout syndrome in students, (4) which was validated for Brazilian Portuguese in 2006. (16) This version has satisfactory psychometric properties attested by factorial analysis and Cronbach's alpha between 0.65 and 0.94. It is an instrument consisting of 15 items that measure three dimensions: emotional exhaustion, depersonalization and academic effectiveness in seven-point Likert scale responses (0-6). It is considered that there is an indication of burnout in students who present high scores on emotional exhaustion (≥16 points) and depersonalization (≥11 points) and low scores on academic effectiveness (≥10 points).

Data were analyzed using the Statistical Package for Social Sciences software, version 20.0. Frequencies and percentages were calculated for categorical variables, and measures of central tendency and dispersion for continuous variables. Three multiple linear regressions were performed by the stepwise forward method, considering the dimensions of burnout syndrome as dependent variables and all independent variables (sociodemographic and academic characteristics) that presented p≤0.20 in the bivariate analysis by simple linear regression. In each model the set of variables that best explained the outcome remained, being applied as statistical criteria: (1) to present statistical significance (p <0.05) and (2) to adjust the values of β by at least 10 %.

Following, began the qualitative phase, which included only participants of the quantitative phase that showed indicative for burnout syndrome or who have expressed simultaneously high scores on emotional exhaustion and depersonalization. Students who met these criteria were entered into a draw to organize the sequence of interviews to include at least two students per course series to represent their various moments. These individuals were contacted by telephone and invited to participate in a recorded individual interview, scheduled according to their availability, and there were no refusals. To determine the number of interviewees, the criterion of theoretical saturation of the discourses was used, that is, the interviews were ceased when no new elements were identified to deepen theorization, which occurred with 21 students. The interviews were conducted from November to December 2016, by a trained researcher with previous experience in this collection method. The interviews were based on the following questions: Tell me how you characterize your experience of being a nursing student? Are there course activities that are exhausting or promote exhaustion and overload? What strategies do you use to deal with this? How do you evaluate your involvement and performance in the course?

The sources produced in the interviews were analyzed according to the condition of the Collective Subject Discourse. This methodological framework was adopted because it is indicated for mixed methodological designs and constitutes a way of expressing the thought of a collectivity about a given phenomenon, as if it were a “collective person”.(15) To support the analysis of qualitative data, we used the DSCsoftTM program, which follows the steps: discourse analysis, in which all narratives were analyzed individually; below the Key Expressions were identified, that is, the excerpts of literal transcriptions with the essential content or underlying theories; following this, the Central Ideas were taken from each key phrase, describing them in a synthethic and punctual manner; subsequently, the Anchorages were established, which explicitly manifested the theory adopted. At the end of the analysis, the statements of different interviewees who had the same meaning were gathered in a first-person singular synthesis-speech.(15) Academic effectiveness was reduced from the perspective of nursing students, which are the dimensions of the syndrome elaborated three synthesis speeches: emotional exhaustion, depersonalization and burnout in this population.

This research was conducted in accordance with national and international standards for research involving human subjects, including approval by the Research Ethics Committee (Opinion No. 354,514). All participants signed an informed consent form.

## Results

Among the 114 nursing students in the study, ages ranged from a minimum of 17 to a maximum of 40 years, with a mean of 21.3 ± 3.5 years. Most students were female (89.5%), single (96.5%), childless (92.1%), living with their families (63.2%), leisure activities (53.3%), not working (95.6%) and did not practice physical activity (76.3%).

Regarding the academic characterization, the assignments are offered in the annual series and, thus, the students attended, on average, 7 with extremes in 3 and 14, and the total workload of the assignments attended in the school year varied between 558 and 960 hours, with an average of 783 hours. In addition to the compulsory activities of the course, they devoted an average of 1.8 hours daily to extra-class studies, ranging from zero to 6 hours. It was found that 28.9% received scientific initiation or extension scholarships and 83.3% expressed satisfaction with the course.

Regarding the MBI-SS dimensions, it was found that 76.3% of participants had high emotional exhaustion, 31.6% high depersonalization and 21.1% low academic effectiveness. Aggregating these dimensions, it was found that 10.5% of nursing students had indicative for burnout syndrome, as well as 30.7% concomitantly had high emotional exhaustion and depersonalization and, therefore, were predisposed to develop the syndrome. 

Table 1 shows the multiple linear regression analyzes performed for the three dimensions of the syndrome. The highest emotional exhaustion scores were associated with nursing students without stable marital relationships, who were in the most advanced grades of the course, with the highest workload of signatures and who were dissatisfied with the nursing course. The second model indicated that the higher the student's age, the more advanced the school year and the higher course load, as well as being dissatisfied with the course, the higher the depersonalization scores; whereas undergraduate or extension fellows obtained the lowest scores in this dimension. Regarding academic effectiveness, the more advanced the school year, the greater the workload of assignments attended and the fewer hours of daily extra-class study, the lower the scores presented by students in this dimension.

**Table 1:** Multivariated models of burnout dimensions between nursery students.

Models	β (Beta)	β IC95%	p-value
Emotional Exhaustion (R=0.420; R^2^=0.176)*			
School year	4.061	1.462; 6.660	0.003
Total workload of assignments attended	0.032	0.014; 0.051	0.001
Satisfaction with the course	-2.812	-0.011; -5.613	0.049
Stable marital relationship	-4.612	-9.953; 0.729	0.090
Depersonalization (R=0.562; R^2^=0.316)*			
School Year	4.487	2.238; 6.736	<0.001
Total workload of assignments attended	0.031	0.016; 0.046	<0.001
Satisfaction with the course	-4.049	-1.704; -6.394	0.001
Age	0.251	-0.020; 0.523	0.069
Scientific or extension scholarship holder	-1.633	-0.294; -3.561	0.096
Academic effectiveness (R=0.326; R^2^=0.106)*			
School Year	-3.527	-6.253; -0.802	0.012
Total workload of assignments attended	-0.020	-0.039; -0.001	0.042
Hours of extra-class daily study	0.799	-0.033; 1.631	0.059

**Multiple correlation coefficient and coefficient of determination, respectively

The empirical material produced by the 21 interviews was grouped into three summary discourses, which allude to the dimensions of burnout syndrome among students: emotional exhaustion, depersonalization and reduced academic effectiveness, which allowed a better understanding of how the academic context in which they are inserted. may contribute to the development of the syndrome.

Emotional Exhaustion. In this collective discourse, it was evidenced that the nursing students perceived the daily demands of the course as intense, producing overload and physical and emotional exhaustion, as well as indicating symptoms of physical and mental illness: *I feel extremely exhausted and overloaded! The workload is very high, almost without breaks and there are many activities required by the course and the teachers: during the day are theoretical and laboratory classes; I should study at night, because I take several subjects [signatures] and each one requires something different: assignments, seminars, monitoring and tests, but I get home extremely tired physically and emotionally, I have no courage to do anything else, the only will I have is to go to my room and rest. When you have practical classes in different sectors of hospitals and basic health units is even worse, we can not go wrong, because we are dealing with people! I have to adapt to the routine of the unit, develop an intense workload, deal with certain situations that I'm unprepared, and teachers always charge what I saw in theory and other subjects [signatures]. The further the course, the more is required. Although it is good for my training, some teachers act rudely, arrogantly and derogatory, making it difficult for me to perform. All this affects my health, I often have headaches, muscle pain, insomnia, fatigue, stress and irritation.* (Collective Subject Discourse 1)

Depersonalization. It can be seen in this discourse the student's distancing from all the activities of the course, revealing that the demotivation was linked to the feeling of exhaustion and overload as a defensive attitude, to distance yourself from what causes you suffering: All this routine of the course makes me very unmotivated, because I don't have time to eat properly, for leisure and to do the things I like. Honestly, I have no desire to go to the classes of some subjects [signatures], as relevant as they are, I do not want to hear or study them; so I manage absences not to fail, but in all classes I get late. *The practices are a reality shock, made me realize how bad the health system is, there are not enough materials and resources to serve the patient well. That holistic care and according to the principles of SUS is only in theory. Nor did I imagine the size of the suffering of others, patients and their families, at first I cried every day when I remembered the scenes, now I try not to get involved, it is a lot of suffering. Some teachers put a lot of pressure when you are in the health service and I don't react very well to the pressure, I didn't feel like going, when I came home and I cried, and it made me rethink about the profession, because I get discouraged when I think nursing is an undervalued and underpaid profession. So I don't know if I want this for myself for the rest of my life.* (Collective Subject Discourse 2)

Reduced Academic Effectiveness. It stands out in this speech-synthesis that the reduced academic effectiveness was related to the incompetence feeling, in which the student is frustrated because he believed could have better performance, but can not get involved again with their studies due to his exhaustion and discouragement: *The academic environment is very competitive and this is encouraged by the teachers. I feel incompetent for not getting good grades, pass without final exams and to perform well in clinical practice. I can not get a scholarship of scientific initiation, because these things are for the best students, those proactive. In addition, teachers motivate only good students, people who have more difficulty seem to be invisible or treated as a lost and incapable case. Still, I feel guilty when I see that I could have studied more, but I was carried away by fatigue. The health sector is very complex and makes me anxious not to be a good student, to perform poorly and, consequently, not to be a good nurse.* (Collective Subject Discourse 3)

## Discussion

Sociodemographic data of the participants of this research regarding gender, marital status and absence of offspring were also observed in other studies, which identified a trend of female activity in the profession, as well as these students prioritized professional training and financial stability, for later family formation, as it can be an arduous task for women to reconcile full-time academic activities and personal life.(17,18) It was found that 10.5% of participants in this study had indicative for burnout syndrome, a result that was lower than those obtained by investigations conducted with nursing students in Brazil and abroad, which ranged from 24.7% to 41%.(7,8) These findings confirm that the syndrome can affect individuals still in the process of professional education, which may result in newly graduated nurses with less mastery of occupational tasks, less use of research in daily clinical practice, with the intention of leaving the profession,(7) less empathic and inattentive to the needs of health service users, which may interfere with the quality of care provided. Considering this context, it is necessary for higher education institutions to implement actions to promote the mental health of their students, through a proactive approach to stressors and coping mechanisms,(19) because health and well-being of students are strongly influenced by the organizational context.(20) In this sense, there is an international initiative "Healthy Universities" in which universities are committed to promoting the health and well-being of students and staff through an organizational culture that people feel valued, respected, heard, able to contribute and share their opinions.(13) 

Due to the nature of the sequential explanatory mixed research design, the qualitative findings supported the quantitative findings, but also provided contextual meanings. It was shown that 76.3% of respondents had high emotional exhaustion. This is the first dimension of the syndrome to manifest itself, it reflects the lack of energy and psychological resources to deal with the academic overload experienced and can have harmful repercussions for professional education, as it relates to the decline of mental health and interferes with the ability of individual concentration and learning.(6,7,18)

In this study, emotional exhaustion was associated with single students, who were in the most advanced grades of the course, with the largest workload of signatures and were dissatisfied with the course. In addition to corroborating these findings, the speech-synthesis elucidated that the sources of physical and mental overload came from the theoretical and practical classes, the volume of academic work, readings and assessments, which accompanies them day by day. They also mentioned the emotional and physical overload present in the practice of care and that they are not psychologically prepared to face such situations, causing their daily life to be marked by somatic symptoms peculiar to exhaustion and feelings of doubt, disappointment, anxiety, fear, sadness, anger and anguish. These informations are important because it shows students' exposure to long periods of academic activity, particularly in relation to the perceived workload and also the emotional demands of working with people who are ill. The study indicated that it is essential to reduce unnecessary academic overload and help students develop skills to better manage their time. However, it was also reflected that the hours invested in profesional training are fundamental and suggests that the courses should be well designed so that the student's long hours of study are not perceived as somewhat monotonous and unimportant.(19)

Depersonalization is an adaptive psychological response in which the student moves away from the study because it causes suffering and overload,(18) and in this study it was found that the older the student, the more advanced the school year, the greater the course load, being dissatisfied with the course, the higher the depersonalization scores. These results are similar to those found in the occupational environment of nurses, where younger and less satisfied nurses have higher levels of depersonalization, due to the transition period between their idealistic expectations and daily practice.(21)

It was identified that a scholarship of scientific initiation or extension was a protective factor not to distance themselves from the course. The participation of academics in research and extension projects promotes the strengthening of their identity and professional autonomy, and forms a more critical, reflective nurse, committed to the care provided, and better prepared to work in different areas of professional practice(22) and thus, this student gets much more involved with his studies. The distancing of the study was also related to the hard work process of nurses, which is full of physical and psychological demands, complex interpersonal relationships, lack of recognition, autonomy and devaluation of the class, and daily living with suffering, which is largely reported in the literature.(23,24) Thus this student sees no satisfactory reward for his present effort and expresses his discontent with the course and desire to abandon it.

Finally, the low academic effectiveness was also accentuated in the more advanced school years, with the higher course load, which resulted in fewer hours of extra-class daily study. It was found that the students felt guilty for not being able to study anymore and expressed feelings of incompetence, impotence and inferiority when compared with their peers. Furthermore, the role attributed to the teacher as (dis)motivators in this process was verified, which was already observed in a research carried out in Finland, which also showed that a positive interpersonal relationship between teacher-student promotes self-confidence and motivation in the student, in addition to a more effective and less stressful learning process.(25) In several higher education institutions, the thinking that student health and engagement is the exclusive responsibility of the individual and not of the university still predominates, so it does not value, respect, listen and invest in the improvement of the student who has a disinterested posture. This paradigm shift is necessary, and should start from top management through institutional policies

Limitations include the use of self-report measures that are prone to socially desirable responses, the cross-sectional design, in which causalities cannot be inferred, as well as the cross-sectional bias of the healthy student, because those who developed the syndrome may be off course, which would increase the prevalence. The results obtained cannot be generalized, since they portray the context experienced by nursing students of a Brazilian public university. However, this study provides a deeper understanding of the complex nature of this phenomenon, as well as support for university managers and teachers to develop effective strategies to promote their students' mental health and well-being and, consequently, to increase engagement and student success.

The mixed method study made it possible to analyze the sample in depth and in full, because despite the quantitative result of students with indicative for burnout syndrome being lower than the national standard reported in the literature for nursing students, the qualitative approach can demonstrate the weakness in the coping methods, mental suffering and frustration related to self-collection in the face of academic life. Therefore, higher education institutions must adhere to management models with emotional support policies, through a student assistance program that includes biopsychosocial aspects, especially management strategies and coping with academic stressors.
